# Evaluation of pre-dilution combined with optical/fluorescent platelet counting for correcting pseudothrombocytopenia

**DOI:** 10.3389/fmed.2026.1855339

**Published:** 2026-07-10

**Authors:** Lin Xiong, Xingguo Pan, Mengyun Peng, Tianwen Gan, Chengmin Deng, He Zha, Ya Wang

**Affiliations:** 1 Department of Laboratory Medicine, The First People's Hospital of Zunyi (The Third Affiliated Hospital of Zunyi Medical University), Zunyi, China; 2 Scientific Research Center, The First People's Hospital of Zunyi (The Third Affiliated Hospital of Zunyi Medical University), Zunyi, China

**Keywords:** EDTA-dependent platelet aggregation, fluorescent platelet count, hematology analyzer, optical platelet count, pseudothrombocytopenia, sample pre-dilution

## Abstract

**Introduction:**

Pseudothrombocytopenia (PTCP), a common artifact in hematology, often leads to misdiagnosis and unnecessary interventions, with EDTA-dependent PTCP being the most frequent. Current corrective methods are limited by inefficiency and the risk of recurrence.

**Methods:**

This study evaluated an integrated strategy—1:7 pre-dilution of fresh EDTA blood with optical/fluorescent platelet counting (PLT-O/PLT-F)—to correct PTCP caused by various factors. A total of 21 PTCP samples were analyzed using the impedance platelet counting method (PLT-I), conventional PLT-O/PLT-F, and pre-diluted PLT-O/PLT-F modes on Mindray BC-7500 CS and Sysmex XN20 analyzers, with manual counting (PLT-M) as the reference. Assessments included anti-interference efficacy: aggregation interference rejection (AIR), outlier rate (OR), and accuracy: recovery rate (RR), mean bias rate (MBR), root mean square error (RMSE), non-parametric bias analysis, and correlation/agreement tests.

**Results:**

Pre-dilution significantly improved anti-interference: DIL BC7500 PLT-O showed the highest AIR (95.24%) and the lowest OR (19.05%). Conventional modes showed significant negative bias, while pre-diluted modes eliminated bias, with RR within an acceptable range (90–110%) and low RMSE (23.15%). Non-parametric analysis showed no significant difference between DIL PLT-O/F and PLT-M (*p* > 0.05). Correlation and agreement were strong, with slopes near 1 and minimal bias. Bland–Altman analysis confirmed DIL BC7500 PLT-O had the smallest bias and best agreement with PLT-M.

**Discussion:**

Study limitations include a small sample size, single-center data, and testing only two analyzers. In conclusion, 1:7 pre-dilution with PLT-O/PLT-F effectively reduces platelet aggregation in PTCP, improves accuracy, anti-interference, and consistency, and offers a pilot solution for laboratory PTCP management. Further multicenter, large-scale studies are needed for validation.

## Introduction

Pseudothrombocytopenia (PTCP) is a relatively common phenomenon in clinical hematology analysis. It can be triggered by various clinical conditions—including viral infections, autoimmune diseases, sepsis, thrombotic and cardiovascular diseases, heparin-induced thrombocytopenia (HIT), and liver disorders (1). These diverse causes induce PTCP to varying degrees. This may lead to prolonged test result turnaround, repeated blood sampling, and, if not promptly identified, diagnostic errors and unnecessary or even invasive investigations. Unwarranted clinical consequences may follow, including potentially life-threatening events (e.g., inappropriate corticosteroid therapy, platelet transfusion, or splenectomy) (2). Among blood and platelet donors, PTCP incidence ranges from 0.01 to 0.2% (2–4), suggesting a higher prevalence in patient populations.

The causes of pseudothrombocytopenia are multifactorial, mainly due to platelet aggregation or agglutination associated with anticoagulant use or immune-mediated factors (1, 5, 6). EDTA-dependent PTCP is a common cause, as EDTA is routinely used in blood collection tubes; the incidence of EDTA-PTCP ranges from 0.07 to 0.21% (7, 8).

Currently, EDTA-dependent pseudothrombocytopenia can be retested with citrate or other anticoagulants. However, reports of PTCP caused by various anticoagulants have increased. Non-anticoagulant-dependent PTCP, mediated by antibodies unrelated to anticoagulants, is also common. Thus, PTCP has diverse causes.

The conventional impedance platelet counting method (PLT-I), based on the Coulter principle, cannot effectively eliminate PTCP from these factors. In contrast, optical platelet counting assays (PLT-O/PLT-F) use fluorescent dyes that bind intracellular RNA and DNA (9–12). Flow cytometry then measures cellular fluorescence intensity and combines indicators such as cell size and complexity to differentiate and count blood cells, including platelets. These optical methods can minimize interference from other cells and platelet types (13). Some manufacturers have integrated platelet dissociation into automated analyzers to further increase accuracy (14, 15). Used together, these approaches improve the reliability of platelet counts (16, 17). Other interventions—such as smear correction, specimen pre-warming, using multiple anticoagulants, and chemical deaggregating agents (18–26)—are less effective.

PTCP is a complex phenomenon affected by multiple confounding factors, including counting methodologies and pre-analytical variables (27). To address this in clinical practice, our research team established a comprehensive strategy: pre-dilution of fresh EDTA-anticoagulated blood specimens combined with PLT-O/PLT-F detection technology, which manages PTCP induced by different pathogenic mechanisms. This study summarizes the integrated protocol and highlights its benefits, including minimizing the risk of diagnostic errors, reducing unnecessary repeat testing, improving platelet count accuracy, and enhancing reliability across various clinical scenarios. We then discuss the protocol’s anti-interference capability, detection accuracy, concordance, robustness, error characteristics, and systematic bias in detail.

## Patients and samples

A total of 21 blood samples were collected from patients with pseudothrombocytopenia during routine result review at the Department of Laboratory Medicine, First People’s Hospital of Zunyi, between June 2024 and March 2025. Platelet aggregation was initially detected by automated hematology analyzers in routine complete blood count testing, and this abnormal finding was further confirmed via microscopic examination of peripheral blood smears. One tube of EDTA-anticoagulated whole blood was collected from each patient for laboratory rechecking. An aliquot of the fresh whole-blood specimen was diluted with Sysmex DCL diluent at a 1:7 ratio to yield a final volume of 1.2 mL for subsequent experimental analyses.

### Instruments and reagents

Two automated hematology analyzers were routinely used in the clinical laboratory: Mindray BC-7500 CS (Mindray Co., Ltd., Shenzhen, China) with its corresponding reagents, and Sysmex XN20 (Sysmex Corporation, Kobe, Japan) along with the SP10 Automated Blood Smear Preparation Apparatus, matched reagents, and quality control materials. For manual platelet (PLT) counting, a Neubauer Hemocytometer (QiuJing Co., Ltd., Shanghai, China), Wright-Giemsa stain solution (Beisuo Co., Ltd., Zhuhai, China), and Olympus BX51 optical microscope (Evident Corporation, Tokyo, Japan) were employed. EDTA anticoagulant tubes (Becton, Dickinson and Company, United States) were used for blood specimen collection, and Sysmex DCL Dilution Solution (Sysmex Corporation, Kobe, Japan) was utilized for sample dilution.

### Methodology

All collected samples were initially analyzed using the impedance channel of either the Mindray BC-7500 CS or Sysmex XN20 analyzer. If the “3R” recheck rule was triggered and a “platelet aggregation” alarm was generated (28), retesting was immediately performed using the PLT-O channel (for BC-7500 CS) or the PLT-F channel (for XN20), followed by a blood smear examination. When platelet aggregation was confirmed by microscopic observation, a new blood sample was collected using an EDTA-K2 anticoagulant tube. Within 10 min of collection, the well-mixed specimen was diluted with Sysmex DCL diluent at a 1:7 ratio to a final volume of 1.2 mL, and the diluted specimen was then subjected to subsequent testing.

The re-collected EDTA-anticoagulated whole blood samples and pre-diluted samples were analyzed for platelet counts using the PLT-O channel (BC-7500 CS) and the PLT-F channel (XN20), respectively. For manual direct platelet counting (PLT-M), the re-collected EDTA-anticoagulated samples were diluted in ammonium oxalate solution to lyse red blood cells. Two experienced laboratory technicians performed PLT-M using phase-contrast microscopy with modified bovine platelet counters independently. The mean value of the two counts was recorded as the PLT-M result for each specimen. If the relative difference between the two manual counts exceeded 20%, the entire manual counting procedure was repeated to ensure accuracy.

### Statistical analysis

Statistical analysis and difference plotting were performed using Analyse-it Ultimate Edition 5.80.2 (Analyse-it Software, Ltd., Leeds, United Kingdom). Measurement data were expressed as the mean ± standard deviation (X ± SD). A two-tailed *p* value <0.05 was considered statistically significant.

## Results

Results of PLT counts by different methods are shown in Supplementary Table S1.

### Anti-interference effectiveness

The effectiveness of anti-interference was directly measured by the ability of different methods to overcome platelet aggregation interference.

#### Aggregation interference rejection

AIR was defined as the percentage of samples satisfying two criteria: “platelet count by pre-dilution PLT-O/F method by PLT-M method” and “platelet count by pre-dilution PLT-O/F method ≥ 80% of platelet count by PLT-M method” [CLSI EP07-3rd Edition] (29), calculated as [(Number of samples meeting the above two criteria)/Total number of samples] × 100%, as shown in Table 1. The AIR values for XN20 PLT-F, BC7500 PLT-O, diluted (DIL) XN20 PLT-F, and DIL BC7500 PLT-O were 23.81% (5/21), 61.90% (13/21), 85.71% (18/21), and 95.24% (20/21), respectively. Under the DIL mode, the BC7500 PLT-O method exhibited an AIR that was 9.53 percentage points higher than that of the XN20 PLT-F method.

**Table 1:** Evaluation form for interference resistance efficacy.

Method	Interference rejection efficiency (%)	Outlier rate (%)
XN20 PLT-I	N/A	100 (21)
BC7500 PLT-I	N/A	100 (21)
XN20 PLT-F	23.81 (5)	90.48 (19)
BC7500 PLT-O	61.90 (13)	71.43 (15)
DIL XN20 PLT-F	85.71 (18)	47.62 (10)
DIL BC7500 PLT-O	95.24 (20)	19.05 (4)

N/A, not Applicable.

#### Outlier rate

OR is a negative parameter measuring the accuracy and reliability of a detection method. It was defined using the formula: [(Total number of samples − Number of samples with PLT-I/O/F counts within the acceptable limit of 80–120% relative to PLT-M counts)/Total number of samples] × 100% [CLSI EP09C-3rd Edition] (30), as shown in Table 1. The ORs for XN20 PLT-I, BC7500 PLT-I, and XN20 PLT-F were all 100% (21/21). For BC7500 PLT-O, DIL XN20 PLT-F, and DIL BC7500 PLT-O, the ORs were 71.43% (15/21), 47.62% (10/21), and 19.05% (4/21), respectively. Notably, the ORs of the PLT-O/F channels under pre-dilution mode (47.62 and 19.05%) were lower than those under routine mode (90.48 and 71.43%).

### Accuracy and deviation evaluation

Accuracy and deviation evaluation were performed to assess the correctness of measurement results and systematic deviation indices.

#### Recovery rate

RR is an intuitive indicator for evaluating the reproducibility of a detection method in measuring true platelet concentration and assessing accuracy. It was calculated as the overall percentage value (mean ± standard deviation) by comparing individual PLT-O/F counts with PLT-M counts [CLSI EP09C-A3 Edition] (30), with an acceptable range of 90–110%, as shown in Table 2.

**Table 2:** Accuracy and deviation evaluation.

Method	Recovery rate (%)	Mean bias rate (%)	Percentage of median bias (interquartile range, %)	Root mean square error (%)
XN20 PLT-I	N/A	−71.09 ± 25.04	−78.90 (−82.49, −59.69)	75.17
BC7500 PLT-I	N/A	−72.06 ± 23.22	−78.74 (−82.63, −61.49)	75.54
XN20 PLT-F	49.5 ± 26.28	−50.50 ± 26.29	−51.86 (−62.76, −38.24)	56.93
BC7500 PLT-O	80.26 ± 18.55	−19.74 ± 18.56	−16.15 (−28.39, −11.09)	27.09
DIL XN20 PLT-F	96.21 ± 22.84	−3.79 ± 22.85	−2.08 (−14.44, 6.87)	23.15
DIL BC7500 PLT-O	99.19 ± 13.74	−0.81 ± 13.75	0.34 (−7.22, 5.60)	13.77

N/A, not Applicable.

#### Mean bias rate

MBR is a metric that quantifies the average systematic discrepancy between test results and the reference method (PLT-M), calculated as [(PLT-O/F count − PLT-M count)/PLT-M count] × 100%, as shown in Table 2.

#### Root mean square error

RMSE is a comprehensive indicator of measurement variability, calculated as the square root of the mean of squared differences between PLT-O/F counts and PLT-M counts [sqrt (*Σ*(PLT-O/F count − PLT-M count)^2^/n)].

### Non-parametric bias estimation

#### Wilcoxon test and Hodges-Lehmann location shift estimator

As shown in Table 3, the first four methods (XN20 PLT-I, BC7500 PLT-I, XN20 PLT-F, BC7500 PLT-O) all exhibited statistically significant position shifts (*p* < 0.0001). The confidence intervals estimated by the Hodges-Lehmann method did not contain 0, confirming a distinct negative shift. The null hypothesis was rejected for all four methods (P 5), indicating statistically significant differences in distribution positions compared to the reference standard (PLT-M).

**Table 3:** Wilcoxon test *t*-statistic and Hodges–Lehmann position offset estimator for six methods/modes.

Method	Wilcoxon test *T* statistic (exact *p*-value)	Hodges-Lehmann shift (Tukey CI)
XN20 PLT-I	0 (<0.0001)	−103.0 (−131.5, −79.5)
BC7500 PLT-I	0 (<0.0001)	−105.0 (−132.0, −82)
XN20 PLT-F	0 (<0.0001)	−73.5 (−101, −51)
BC7500 PLT-O	9 (<0.0001)	−29.0 (−54.5, −15.0)
DIL XN20 PLT-F	97.5 (0.5392)	−3.0 (−26.5, 10.5)
DIL BC7500 PLT-O	99.0 (0.5854)	−3.5 (−10.5, 4.5)

“H0: Δ = 0 The shift in location between the distributions of the populations is equal to 0. H1: Δ ≠ 0 The shift in location between the distributions of the populations is not equal to 0.” Do not reject the null hypothesis at the 5% significance level.

In the DIL mode, the platelet counts obtained by the PLT-O and PLT-F methods showed no statistically significant differences compared to PLT-M. The Hodges-Lehmann estimators indicated minimal typical differences (−3 and −3.5), with confidence intervals containing 0 and symmetrically distributed around 0, suggesting no significant directional or magnitude-related bias.

### Correlation and agreement assessment

#### Spearman’s rank correlation coefficient

First, the Shapiro–Wilk test was performed to assess the normality of PLT-M (microscopic manual counting) results, yielding a test statistic of 0.905 and a significance level (*α*) of 0.044 (P 05). The null hypothesis was rejected, indicating that the sample data did not follow a normal distribution. The sample skewness was 0.831, the kurtosis was −0.262, and the standard error was 14.960. The skewness-to-standard-error ratio was 0.056, suggesting a mild skewness. Consequently, Spearman’s rank correlation coefficient was used to analyze the correlation between methods, evaluating the strength and direction of the monotonic relationship between paired measurements, as shown in Table 4.

**Table 4:** Spearman’s rank correlation coefficient.

[Spearman’s rs (Fisher 95% CI) *p*-value]	XN20 PLT-I	BC7500 PLT-I	XN20 PLT-F	BC7500 PLT-O	DIL XN20 PLT-F	DIL BC7500 PLT-O	Microscopic methods (PLT-M)
XN20 PLT-I	1.0000	0.952 (0.880, 0.981) **< 0.0001**	0.816 (0.585, 0.925) **< 0.0001**	0.511 (0.089, 0.778) **0.0179**	0.309 (−0.155, 0.661) 0.1734	0.412 (−0.037, 0.723) 0.0632	0.365 (0.093, 0.695) 0.1036
BC7500 PLT-I		1.0000	0.860 (0.675, 0.944) **< 0.0001**	0.533 (0.118, 0.789) **0.0129**	0.387 (−0.067, 0.708) 0.0830	0.454 (0.014, 0.747) **0.0387**	0.415 (−0.034, 0.725) 0.0613
XN20 PLT-F			1.0000	0.497 (0.069, 0.770) **0.0220**	0.440 (−0.003, 0.739) 0.0547	0.349 (−0.111, 0.686) 0.1208	0.266 (−201, 0.634) 0.2444
BC7500 PLT-O				1.0000	0.676 (0.337, 0.861) **0.0008**	0.735 (0.433, 0.889) **0.0001**	0.753 (0.465, 0.897) **< 0.0001**
DIL XN20 PLT-F					1.0000	0.786 (0.526, 0.911) **< 0.0001**	0.814 (0.581, 0.924) **< 0.0001**
DIL BC7500 PLT-O						1.0000	0.977 (0.941, 0.991) **< 0.0001**
Microscopic methods (PLT-M)							1.0000

rs = 1 indicates perfect positive correlation, rs = −1 indicates perfect negative correlation, and rs = 0 indicates no correlation. The values of ∣rs∣ ranging from 0.4 to 0.59, 0.6 to 0.79, and 0.8 to 1.0 correspond to moderate, strong, and very strong correlations, respectively. H0: The variables are independent. H1: The variables are not independent. When *p* < 0.05, reject the null hypothesis in favor of the alternative hypothesis at the 5% significance level. Then *p* < 0.05, *p* < 0.01, and *p* < 0.001 are, respectively, considered to be “statistically significant,” “very significant,” and “extremely significant.” Values in bold indicate statistically significant differences between groups.

Strong and extremely significant positive correlations were observed among XN20 PLT-I, BC7500 PLT-I, and XN20 PLT-F. Similarly, DIL XN20 PLT-F, DIL BC7500 PLT-O, and PLT-M exhibited strong and extremely significant positive correlations. In the DIL mode, XN20 PLT-F and BC7500 PLT-O also showed a strong, extremely significant positive correlation. Notably, BC7500 PLT-O demonstrated moderate and significant correlations with XN20 PLT-I, BC7500 PLT-I, and XN20 PLT-F, while displaying strong and extremely significant positive correlations with DIL XN20 PLT-F, DIL BC7500 PLT-O, and PLT-M.

#### Passing-Bablok regression analysis

**Figure 1:**
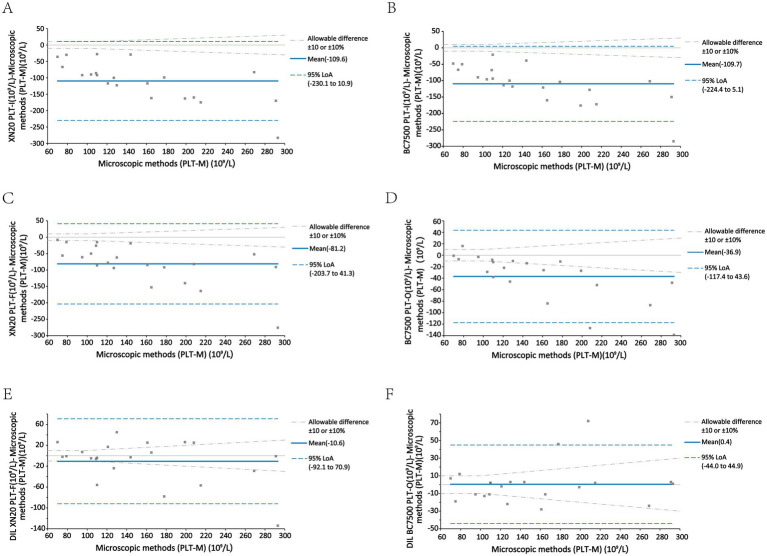
Passing-Bablok regression analysis between 6 detection methods and microscopic methods (PLT-M), (seed: 250909), **(A–F)** XN20 PLT-I, BC7500 PLT-I, XN20 PLT-F, BC7500 PLT-O, DIL XN20 PLT-F, DIL BC7500 PLT-O.

As shown in Figure 1, Passing-Bablok regression analysis was conducted to evaluate bias types and agreement levels between six automated detection methods and the reference method (PLT-M). The slopes of XN20 (1.000) and BC7500 (1.033) in the DIL mode were closest to 1 compared to PLT-M. In the conventional PLT-O/F mode, the slopes of XN20 (0.7468) and BC7500 (0.7143) were both less than 1, indicating proportional bias. Notably, except for BC7500 PLT-O, which had a positive intercept, the intercepts of the other five methods were negative. Detailed evaluation results are as follows:

1. PLT-I mode: A highly significant bias was observed. Both XN20 and BC7500 exhibited significant systematic bias in the PLT-I (impedance method) mode, with a slope point estimate of approximately 0.53 (far below 1), indicating severe proportional negative bias. Measured values were significantly underestimated as PLT-M counts increased. The negative intercept (approximately −40) suggested a constant negative bias.
2. Conventional PLT-O mode: High risk of underestimation persisted. The conventional PLT-O (optical method) mode still exhibited systematic underestimation but was improved compared to the PLT-I mode. The slope point estimates for XN20 and BC7500 were 0.7468 and 0.7143, respectively, indicating proportional negative bias. Notably, only the intercept of BC7500 PLT-O was positive, suggesting a constant positive bias.
3. DIL mode: Highest consistency with PLT-M. Under the DIL PLT-O/F mode, the count results of both instruments showed significantly improved consistency with PLT-M compared to the conventional PLT-O/F mode. The slope point estimates for XN20 and BC7500 were 1.000 and 1.033, respectively (closest to 1), with no significant proportional bias. The intercept point estimates (−2 and −6.54) were closest to 0, and their confidence intervals included 0, indicating no significant constant bias.

In conclusion, among the six methods, the DIL PLT-O/F mode exhibited the best consistency with PLT-M, with regression parameters showing almost no proportional or constant bias. The conventional PLT-O mode showed proportional underestimation, while the PLT-I mode exhibited the most severe underestimation.

#### Weighted Deming regression analysis

Given that the gold-standard PLT-M method also exhibits inherent systematic errors, Weighted Deming Regression analysis was further employed to more rigorously evaluate systematic bias. Consequently, only three methods—BC7500 PLT-O, DIL XN20 PLT-F, and DIL BC7500 PLT-O—could be fitted to the regression equation, as shown in Table 5. XN20 PLT-I, BC7500 PLT-I, and XN20 PLT-F failed to fit the equation. Detailed results are as follows:

1. BC7500 PLT-O: A slight underestimation trend was observed (slope = 0.78), but its slope confidence interval included 1, and the intercept confidence interval included 0, indicating no statistically significant bias. Its prediction accuracy was moderate (Sy.x = 0.3).
2. DIL XN20 PLT-F: An overestimation trend was observed (slope = 1.21), with the widest confidence intervals for slope and intercept and the most significant standard error, indicating higher estimation uncertainty and the lowest prediction accuracy (Sy.x = 0.4).
3. DIL BC7500 PLT-O: The best performance was observed, with a slope (1.14) closest to 1, the smallest intercept, the narrowest confidence interval, and the smallest standard error—indicating the best consistency with PLT-M and the most reliable estimation. Additionally, its weighted Sy.x value was the lowest (0.1), reflecting the highest prediction accuracy.

**Table 5:** Weighted Deming regression analysis between six detection methods and microscopic methods (PLT-M).

Method	Slope, (Slope_LCL, Slope_UCL)	Jackknife SE (Slope)	Inter, (Inter_LCL, Inter_UCL)	Jackknife SE (Inter)	Weighted Sy.x (vertical)
XN20 PLT-I	None	None	None	None	None
BC7500 PLT-I	None	None	None	None	None
XN20 PLT-F	None	None	None	None	None
BC7500 PLT-O	0.7786, (0.4932, 1.0640)	0.1364	−2.827, (−43.60, 37.95)	19.481	0.3
DIL XN20 PLT-F	1.214, (0.5216, 1.905)	0.3306	−42.01, (−138.4, 54.42)	46.07	0.4
DIL BC7500 PLT-O	1.139, (0.9275, 1.351)	0.1011	−21.08, (−48.81, 6.648)	13.249	0.1

#### Bland–Altman plot and analysis

**Figure 2:**
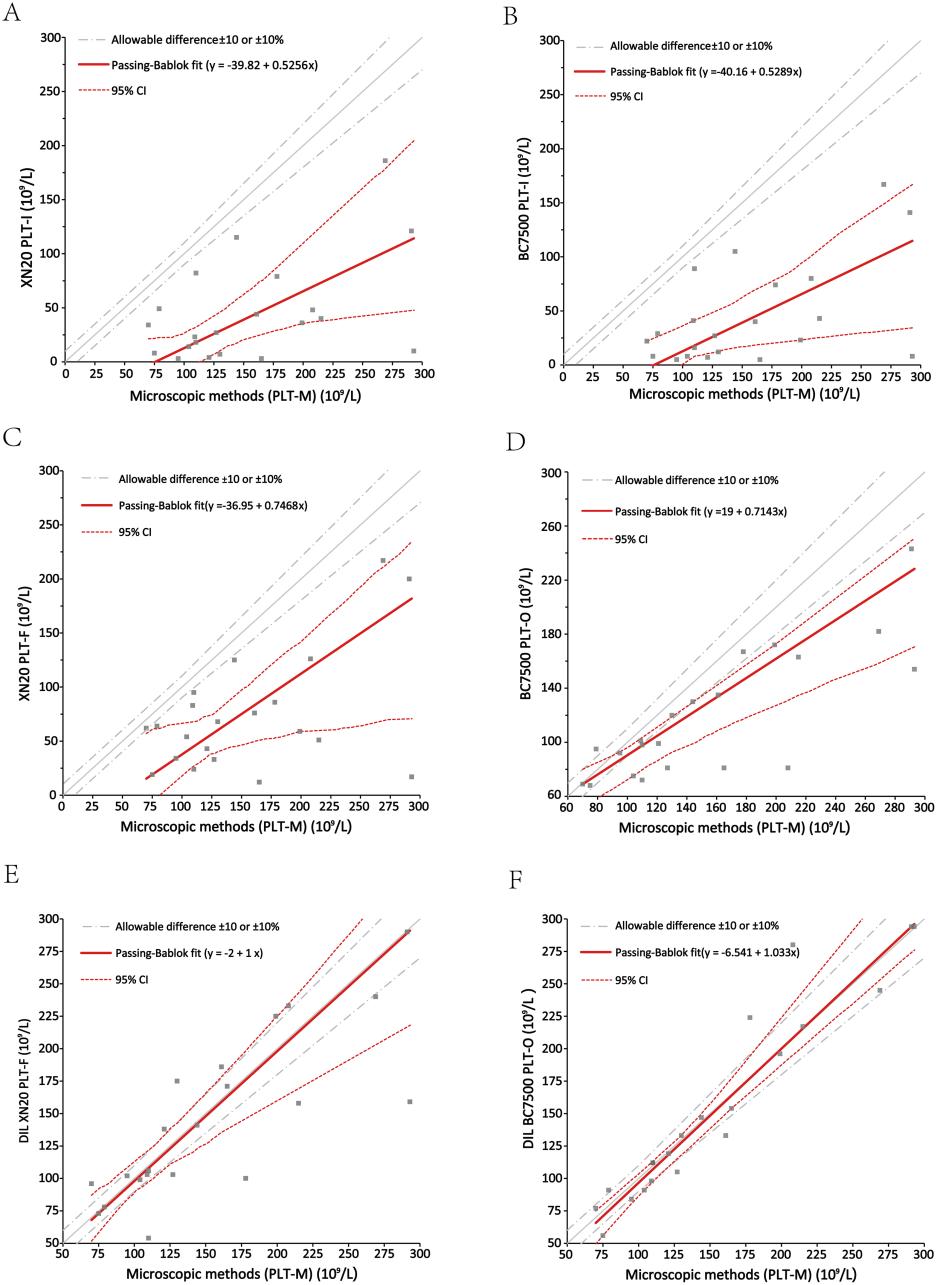
Bland–Altman consistency analysis of six detection methods compared to the microscopic reference method (PLT-M), **(A–F)** XN20 PLT-I, BC7500 PLT-I, XN20 PLT-F, BC7500 PLT-O, DIL XN20 PLT-F, DIL BC7500 PLT-O.

Bland–Altman bias analysis visually presents the absolute differences and agreement limits (LoA) between each detection method and the reference method (PLT-M), as shown in Figure 2.

#### Overall bias analysis

The mean difference for all methods was negative, indicating that most methods systematically underestimated PLT-M counts. This underestimation trend gradually weakened from the PLT-I mode to the DIL mode:

1. PLT-I mode: The most severe underestimation was observed, with average biases for XN20 and BC7500 of approximately −109.7, resulting in an average underestimation of PLT-M of about 110 units.
2. Conventional PLT-O mode: Underestimation remained significant but improved compared to the PLT-I mode. The average biases for XN20 and BC7500 were −81.2 and −36.9, respectively.
3. DIL mode: The smallest bias was observed. The average bias for DIL XN20 PLT-F dropped to −10.6, and the average bias for DIL BC7500 PLT-O was 0.4. The 95% confidence interval (−9.89, 10.75) for DIL BC7500 PLT-O included 0, indicating no remarkable systematic bias.

#### Consistency and dispersion analysis

In conclusion, the Bland–Altman analysis confirmed that DIL BC7500 PLT-O demonstrated the best consistency with PLT-M, with an average bias approaching zero and the least inter-individual variability. In contrast, the PLT-I and conventional PLT-O modes exhibited significant systematic underestimation and high variability, resulting in suboptimal consistency with PLT-M.

## Discussion

Pseudothrombocytopenia (PTCP) is a complex phenomenon driven by endogenous/exogenous factors (e.g., plasma antibodies, anticoagulants) (1), making repeated anticoagulant tube replacement inefficient and clinically impractical. Our team observed both EDTA-K2- and citrate-dependent PTCP in preliminary studies, leading to the development of a combined method: pre-dilution (1:7 ratio within 10 min) of fresh EDTA-anticoagulated samples, followed by PLT-F/O detection. This study elaborates on the protocol and conducts a multidimensional evaluation, with dilution effects on other blood components deemed acceptable (per prior linearity validation) (31).

### Interference rejection efficiency

Per CLSI EP07-3rd Edition (29), effective interference rejection requires an Acceptable Interference Rate (AIR) ≥ 80% (counts ≥ 80% of the gold standard PLT-M). Both DIL XN20 PLT-F and DIL BC7500 PLT-O met this criterion, with pre-dilution improving AIR by 61.9 and 33.34 percentage points, respectively. The BC7500’s inherent interference control (additives, agitation, and physiological temperature maintenance) likely contributed to its baseline performance. For the outlier rate (OR, per CLSI EP09C-3rd Edition (30), acceptable bias range: 87.5–112.5%), only DIL PLT-F/O had an OR (DIL PLT-O performed better). Despite strict acceptable limits (−12.5–12.5%), reference change values RCV:−16.8 (32) and low instrument CV 3.01 confirmed pre-dilution + PLT-F/O significantly enhances anti-interference ability and accuracy.

### Accuracy and deviation assessment

Conventional PLT-I mode showed a severe systematic negative bias, no valid recovery rate (RR), and an RMSE > 75%, failing to reflect true platelet concentrations (15). Undiluted PLT-F/O modes improved performance but did not meet CLSI’s 90–110% RR standard (30, 33), with persistent systematic bias and inter-sample variability (BC7500 PLT-O outperforming XN20 PLT-F). In contrast, diluted PLT-F/O modes achieved qualitative accuracy improvements: all metrics met CLSI standards, systematic bias was largely eliminated, RMSE dropped to 23.15%, and only minor random bias remained. Correlation analysis indicated that bias was primarily systematic, suggesting that future optimization should focus on formula calibration rather than outlier removal (34).

### Nonparametric estimation of systematic bias

Using the Wilcoxon T + Hodges-Lehmann (HL) + Tukey CI approach, non-diluted methods did not reject the null hypothesis (*p* > 0.05), indicating no significant distribution differences from PLT-M. Diluted PLT-F/O modes did not differ (*p* > 0.05), with no statistically significant difference. All methods yielded negative HL estimates (results lower than PLT-M), and the shift degree correlated significantly. Nonparametric tests were chosen for robustness against outliers and non-normal distribution (35–39).

### Correlation and agreement assessment

Shapiro–Wilk testing confirmed non-normal data (40), prompting Passing-Bablok and weighted Deming regression. Passing-Bablok results showed DIL XN20 PLT-F had a median slope of 1 (minimal proportional bias), while BC7500 PLT-O exhibited unique positive constant bias. Weighted Deming regression (accounting for gold standard error) found: (1) BC7500 PLT-O had a slope near 1 (good consistency); (2) DIL XN20 PLT-F (slope 1.214) showed overestimation and unstable correlation; (3) DIL BC7500 PLT-O (slope 1.139) had stable positive correlation and mild overestimation. DIL BC7500 PLT-O also had the smallest Jackknife SE (reliable parameters) and Weighted Sy. x (0.1, high prediction accuracy). Bland–Altman analysis validated the consistency of individual samples, reflecting strict control of the dilution ratio and procedures.

## Limitations and future directions

Key limitations include a small sample size (due to PTCP collection challenges), a limited instrument scope (XN20/BC7500), and single-institution, narrow disease-spectrum samples. Future research should expand the sample size, conduct multicenter/multi-instrument validation, include diverse diseases, optimize dilution ratios, or integrate aggregation risk prediction algorithms.

More notably, Articles 7.2, 6.1, and 6.2 of the CLSI EP09-A3 standard explicitly specify scenarios permitting flexible adjustments to sample size requirements, while clearly distinguishing between performance statements established by manufacturers (with a mandatory minimum requirement of 100 patient samples) and existing statements from clinical laboratory validation institutions (with a recommended minimum requirement of 40 patient samples) (30). When a laboratory conducts only validation without performing new bias assessment evaluations, it may reasonably determine the required sample size based on the corresponding clinical risk assessment results.

Furthermore, Chapter 8 of CLSI EP15-A3, “User Validation and Bias Estimation for Precision,” describes a patient-sample comparison protocol that requires only 20 samples. This protocol is specifically designed for analytes for which patient samples are scarce or difficult to obtain (41). CLSI EP10-A3, “Preliminary Evaluation of Quantitative Measurement Methods,” establishes protocols for preliminary method screening and pre-validation, in which 15 to 25 patient samples are sufficient for exploratory comparative analyses (42).

Regarding China’s domestic regulatory standards, Clause 5.2 of CNAS-GL047:2021 “Guidelines for the Validation of Comparability of Quantitative Test Results in Medical Laboratories” does not mandate the use of 40 samples as a strict threshold. Laboratories may determine an appropriate sample size based on analyte scarcity and clinical risks, provided that the validation protocol is fully documented and clear acceptance criteria are established (43).

Despite its limitations, the pre-dilution + PLT-F/O method has proven to be a simple, cost-effective PTCP correction approach that rapidly provides relatively reliable results for specific specimens. This method streamlines workflows and is valuable for laboratory PTCP management.

## Data Availability

The original contributions presented in the study are included in the article/Supplementary material, further inquiries can be directed to the corresponding authors.
