# Supplementary material for: Evaluation of pre-dilution combined with optical/fluorescent platelet counting for correcting pseudothrombocytopenia
Source: Front Med (Lausanne). 2026 Jul 10;13:1855339. doi: 10.3389/fmed.2026.1855339 (PMC13395694; doi:10.3389/fmed.2026.1855339)
Supplement: Supplementary file 1 [file Table_1.docx]

|  | Department | Bed No. | Clinical Diagnosis | XN20 PLT-I  (10^9^/L) | BC7500 PLT-I  （10^9^/L） | XN20 PLT-F  (10^9^/L) | BC7500 PLT-O  （10^9^/L） | DIL XN20 PLT-F  (10^9^/L) | DIL BC7500 PLT-O  (10^9^/L) | Microscopic methods (PLT-M) (10^9^/L) |
| --- | --- | --- | --- | --- | --- | --- | --- | --- | --- | --- |
| 1 | 3rd Obstetric Ward | Bed 81 | Active labor | 79 | 74 | 86 | 167 | 100 | 224 | 178 |
| 2 | Anorectal Surgery | Bed 32 | Anal fistula | 7 | 12 | 68 | 120 | 175 | 133 | 130 |
| 3 | 1st Orthopedic Ward | Bed 20 | Right knee osteoarthritis | 8 | 8 | 19 | 68 | 73 | 56 | 75 |
| 4 | Rheumatology and Immunology Outpatient |  | Rheumatoid arthritis | 49 | 29 | 64 | 95 | 78 | 91 | 79 |
| 5 | 2nd Neurology Outpatient |  | Neuropathic headache | 44 | 40 | 76 | 135 | 186 | 133 | 161 |
| 6 | Emergency Internal Medicine | Bed 27 | Fatigue | 3 | 5 | 34 | 92 | 102 | 84 | 95 |
| 7 | Breast and Thyroid Surgery | Bed 26 | Thyroid nodule | 40 | 43 | 51 | 163 | 158 | 217 | 215 |
| 8 | Infectious Diseases Outpatient |  | Pulmonary tuberculosis | 115 | 105 | 125 | 130 | 141 | 147 | 144 |
| 9 | 1st Orthopedic Outpatient |  | Right knee arthroplasty | 36 | 23 | 59 | 172 | 225 | 196 | 199 |
| 10 | Respiratory and Critical Care Medicine | Bed 90 | Pulmonary infection | 4 | 7 | 43 | 99 | 138 | 119 | 121 |
| 11 | Hepatic Surgery | Bed 56 | Trauma due to traffic accident | 23 | 41 | 83 | 101 | 103 | 98 | 109 |
| 12 | 3rd Obstetric Ward | Bed 85 | Active labor | 10 | 8 | 17 | 154 | 159 | 294 | 293 |
| 13 | Rheumatology and Immunology Outpatient |  | Rheumatoid arthritis | 14 | 8 | 54 | 75 | 99 | 91 | 104 |
| 14 | Gastrointestinal Surgery | Bed 47 | Rectal cancer | 3 | 5 | 12 | 81 | 171 | 154 | 165 |
| 15 | Otolaryngology | Bed 44 | Right parotid abscess | 48 | 80 | 126 | 81 | 233 | 280 | 208 |
| 16 | Rheumatology and Immunology Outpatient |  | Rheumatoid arthritis | 34 | 22 | 62 | 69 | 96 | 77 | 70 |
| 17 | Neurological Intensive Care Unit (NICU) | Bed 17 | Craniocerebral trauma | 82 | 89 | 95 | 98 | 106 | 112 | 110 |
| 18 | Gastrointestinal Surgery | Bed 53 | Intestinal obstruction | 27 | 27 | 33 | 81 | 103 | 105 | 127 |
| 19 | Urology Surgery | Bed 42 | Right ureteral calculus | 186 | 167 | 217 | 182 | 240 | 245 | 269 |
| 20 | Gastrointestinal Surgery | Bed 75 | Acute appendicitis | 121 | 141 | 200 | 243 | 290 | 294 | 291 |
| 21 | Intensive Care Unit | Bed 10 | Cervical trauma | 18 | 16 | 24 | 72 | 54 | 112 | 110 |

Table S1 Results of PLT counts by different methods
