# Supplementary material for: Evaluation of pre-dilution combined with optical/fluorescent platelet counting for correcting pseudothrombocytopenia
Source: Front Med (Lausanne). 2026 Jul 10;13:1855339. doi: 10.3389/fmed.2026.1855339 (PMC13395694; doi:10.3389/fmed.2026.1855339)
Supplement: Supplementary file 2 [file Table_2.docx]

Table S2. Corresponding reference change value (RCV) of two hematology analyzers

|  | CVI | XN20 | | | BC7500 | | |
| --- | --- | --- | --- | --- | --- | --- | --- |
|  |  | CVA | RCV% Increase | RCV% Decrease | CVA | RCV% Increase | RCV% Decrease |
| Median | 7.3 | 3.01 | 20.1 | -16.8 | 2.87 | 20 | -16.7 |
| Lower CI | 6.6 |  | 18.4 | -15.5 |  | 18.2 | -15.4 |
| Upper CI | 10.2 |  | 28 | -21.9 |  | 27.9 | -21.8 |

Note: CVa: Coefficient of Variation of Analysis, representing the imprecision of laboratory testing methods. CVi: Coefficient of Variation within an individual, representing physiological fluctuations within a healthy individual over a short period (e.g., during the day).
